# Exploring the Mechanisms of *Hydrangea macrophylla* Adapting to Low Light-Induced Ornamental Whitening Through Physiological, Transcriptional, and Metabolomic Analyses

**DOI:** 10.3390/genes17050545

**Published:** 2026-05-02

**Authors:** Wenji Li, Long Guo, Chuanshuai Li, Yao Li

**Affiliations:** 1 School of Design, Chongqing Industry Polytechnic University, Chongqing 401120, China; lichuanshuai1989@gmail.com; 2 College of Landscape Architecture, Sichuan Agricultural University, Chengdu 611130, China; guo_long1013@163.com (L.G.); 15279908429@163.com (Y.L.)

**Keywords:** low light stress, *Hydrangea macrophylla*, ornamental whitening, multi-omics analysis

## Abstract

**Background/Objectives:** To explore the mechanisms of *Hydrangea macrophylla* adapting to low light-induced ornamental whitening, this study established treatments involving normal light (CK, 200 μmol·m^−2^·s^−1^), moderate low light (L1, 100 μmol·m^−2^·s^−1^), and severe low light (L2, 20 μmol·m^−2^·s^−1^). **Methods:** Meanwhile, physiological indicators, including growth, photosynthesis, and antioxidant activity, were assessed, alongside transcriptomic and metabolomic analyses. **Results:** Results indicate that L1 increased the proportion of leaf whitening area while maintaining plant growth (crown width, biomass), photosynthetic efficiency comparable to CK, and superior to L2. Concurrently, L1 activated a coordinated antioxidant defence system, namely by increasing the activity of key enzymes (e.g., SOD, GR) and the accumulation of protective metabolites (e.g., soluble proteins, total phenolics and total flavonoids), thereby minimising oxidative damage (low MDA). Multi-omics analyses revealed that L1 specifically activated these networks associated with carbon assimilation, energy metabolism, secondary metabolite synthesis, and hormone signalling, indicating a systemic molecular mechanism towards enhanced defence. **Conclusions:** In summary, moderate low light triggers a synergistic molecular network involving enhanced antioxidant defences and secondary metabolism, enabling *H. macrophylla* to maintain overall physiological homeostasis and healthy growth while exhibiting ornamental whitening phenotypes, thereby revealing a unique aesthetic adaptation mechanism to environmental stress.

## 1. Introduction

*Hydrangea macrophylla* (*H. macrophylla*) is renowned for its rich floral colours, which can be regulated by pH and metal ions, making it an important research subject in horticultural ornamentation and plant science [1,2]. However, compared to the in-depth study of the *H. macrophylla* flower variety, the intrinsic physiological and molecular mechanisms of leaf colour variation (e.g., ornamental whitening or mottling) arising under specific environmental conditions remain unclear. Leaf colour serves as a core trait for assessing both the ornamental value and physiological condition of plants, with its abnormal variations frequently linked to photosynthetic function, chloroplast development, and metabolic equilibrium [3,4]. In horticultural practice, stable and aesthetically pleasing leaf colour variations (e.g., variegated cultivars) hold high commercial value [5,6,7]. Preliminary research observed an intriguing phenomenon: low-light treatment induced increased whitening regions on *H. macrophylla* leaves, marginally enhancing their visual appeal. Therefore, unravelling the mechanisms behind this phenomenon not only aids in understanding the plant’s environmental adaptation strategies but also paves the way for novel and promising technical approaches to actively “shape” ornamental traits through non-genetic means—namely, environmental regulation.

Light serves as both the energy source and core signal for plant life processes. As a common abiotic stressor, low light forces plants to strike a delicate balance between energy capture, carbon allocation, and defence against adversity [8,9]. Typical low-light adaptation strategies encompass morphological adjustments (e.g., increasing leaf area) and photo-physiological optimisations (e.g., enhancing the chlorophyll b/a ratio) [9,10,11]. However, persistent or intense low-light stress can lead to reduced photosynthetic efficiency and increased reactive oxygen species (ROS) accumulation, potentially triggering photo-oxidative damage and chlorophyll degradation, resulting in leaf yellowing or whitening [12,13]. To counter oxidative stress, plants have evolved a defence system comprising enzymatic pathways (e.g., enhanced SOD, CAT, POD, GR, and PAL activity), and non-enzymatic substances (e.g., increased levels of proline, glutathione, ascorbic acid, and secondary metabolites including phenolic and flavonoid compounds) [14,15]. Among these, proline functions concurrently as a permeation regulator, free radical scavenger, and signalling molecule; alterations in soluble sugars and soluble proteins reflect the redistribution of carbon and nitrogen metabolism; while secondary metabolites such as total phenolics and total flavonoids, serving as crucial antioxidants, often accumulate in parallel with enhanced stress resistance [16,17]. Notably, under certain conditions, specific light environments can induce the synthesis of pigments such as anthocyanins, resulting in ornamental coloured foliage [18,19], indicating that light signals interact in complex ways with plant pigment metabolism and stress response networks.

Furthermore, to gain deeper insight into its underlying mechanisms, it is essential to explore the levels of gene expression regulation and dynamic changes in metabolites. Transcriptomics comprehensively reveals which genes are specifically induced or suppressed under particular treatment conditions, thereby precisely identifying key response pathways and regulatory hubs such as photoreception, chlorophyll biosynthesis and degradation, ROS sensing and signalling, and secondary metabolic pathways [14,20,21]. Metabolomics can systematically analyse the types and quantitative changes in intracellular small-molecule metabolites, particularly primary and secondary metabolites associated with pigment synthesis, antioxidant defence, energy metabolism, and osmotic regulation [14,22,23]. Integrated physiological, transcriptomic, and metabolomic data analysis provides a clear explanation of how external treatments drive alterations in specific metabolic pathways by regulating the expression of core genes, ultimately leading to phenotypic manifestations and corresponding changes in physiological and biochemical indicators [17,24]. This integrated physiological and multi-omics research strategy has become a widely applied tool for deciphering the mechanisms underlying complex biological traits [25].

Therefore, this study utilised *H. macrophylla* as the experimental material, establishing comparative treatments under normal light (CK, 200 μmol·m^−2^·s^−1^), moderate low light (L1, 100 μmol·m^−2^·s^−1^), and severe low light (L2, 20 μmol·m^−2^·s^−1^), focusing on the ornamental whitening phenotype. By dynamically monitoring physiological indicators related to growth, photosynthesis, and antioxidation, and combining this with transcriptomic and untargeted metabolomic analyses, this study aimed to clarify how moderate low light induces plant whitening while maintaining overall physiological health at the physiological and molecular levels. This study presents a systematic “stress–adaptation–aesthetics” mechanistic model, providing a theoretical basis for understanding plant environmental adaptability and developing new horticultural cultivation techniques.

## 3. Results

### 3.1. Effect of Low-Light on Leaf Whitening Area Formation

**Figure 1:**
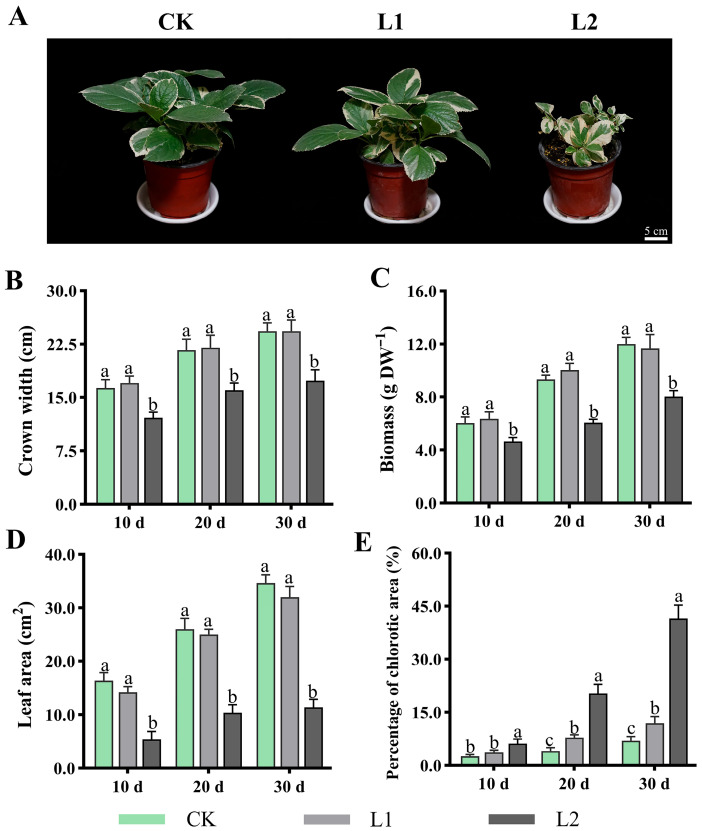
Effects of low light on *H. macrophylla* growth and leaf whitening area formation. (**A**) The overall growth status of the plant after treatment for 30 d. (**B**) Crown width. (**C**) Biomass. (**D**) Leaf area. (**E**) Proportion of whitened regions. Different lowercase letters denote statistically significant differences (*p* < 0.05) among treatments; data values are mean ± SD.

As shown in Figure 1, following treatment under different light intensity conditions, the plants exhibited significant differences in both growth phenotypes and physiological indicators. For growth phenotypes (Figure 1A), plants in the CK and L1 groups exhibited healthier morphology throughout the 30 d experimental period, displaying normal leaf extension and deeper green colouration, with a slight increase in L1-induced whitening regions. In contrast, plants in L2 showed marked growth inhibition and wilted leaves. Quantitative analysis revealed no significant differences in crown width (Figure 1B), biomass (Figure 1C), or leaf area (Figure 1D) between the CK and L1, though all were significantly higher than in the L2. Furthermore, the proportion of whitened regions was higher in L1 than in CK, and both were significantly lower than in L2, for example, after 30 d of treatment: L1 at 11.9%, CK at 6.83%, and L2 at 41.5% (Figure 1E).

### 3.2. Effects of Low Light on Leaf Photosynthetic Systems

**Figure 2:**
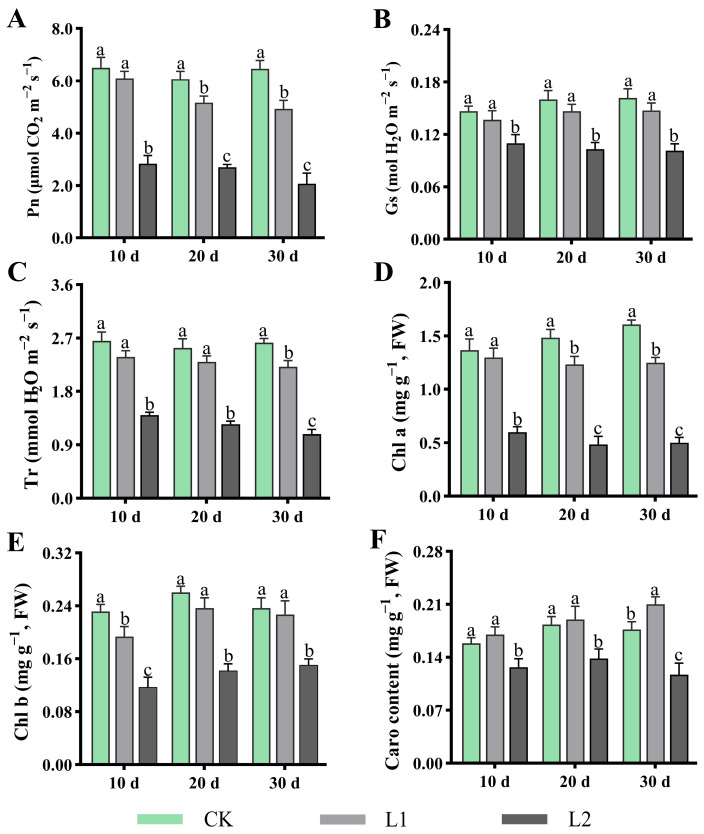
Effects of low light on *H. macrophylla* photosynthetic systems. (**A**) Pn (photosynthesis rate). (**B**) Gs (stomatal conductance). (**C**) Tr (transpiration rate). (**D**) Chlorophyll a. (**E**) Chlorophyll b. (**F**) Carotenoids. Different lowercase letters denote statistically significant differences (*p* < 0.05) among treatments; data values are mean ± SD.

Photophysiological responses and pigment content variations indicate that L1 exhibited no difference compared to CK, but significantly outperformed L2, demonstrating its distinct advantage in maintaining plant photosynthetic function (Figure 2). Specifically, as shown in Figure 2A–C, throughout the entire experimental period, the Pn, Gs, and Tr of L1 showed no difference compared to CK, but remained significantly higher than those of L2. For instance, after 10 d of treatment, the Pn value of Group L1 reached 6.1 μmol CO_2_ m^−2^ s^−1^, representing 0.93 times that of CK and 2 times that of L2. Meanwhile, compared with L2, L1 exerted a lesser effect on photosynthetic pigment accumulation (Figure 2D–F). Specifically, the contents of Chl a, Chl b, and Caro were significantly higher in L1 than in L2, while exhibiting minimal differences relative to CK. In addition, there were no significant differences in Fv/Fm, ΦPSII, qP and ETR between L1 and CK, while these parameters were significantly higher in L2 (*p* < 0.05). qN increases with increasing light intensity, with qN values following the order L2 > L1 > CK (Table 1). Conclusively, by maintaining photosynthetic efficiency, L1 enables the plant’s photosynthetic apparatus to operate efficiently in response to environmental changes.

### 3.3. Effects of Low Light on Leaf MDA and Antioxidant Enzyme Activity

**Figure 3:**
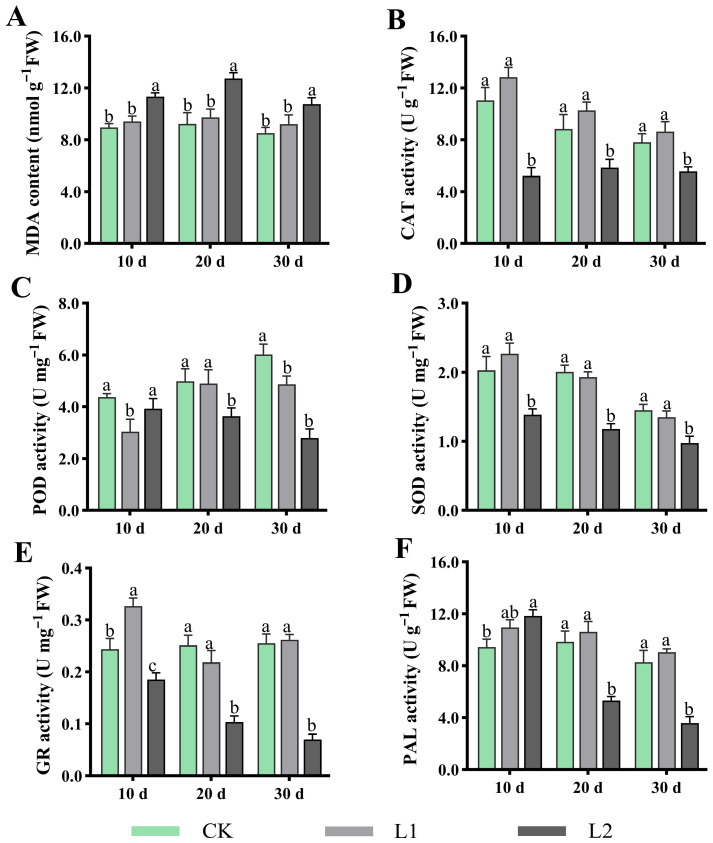
Effects of low light on *H. macrophylla* leaf MDA and antioxidant enzyme activity. (**A**) Malondialdehyde content (MDA). (**B**) Catalase activity (CAT). (**C**) Peroxidase activity (POD). (**D**) Superoxide dismutase activity (SOD). (**E**) Glutathione reductase activity (GR). (**F**) Phenylalanine ammonia lyase activity (PAL). Different lowercase letters denote statistically significant differences (*p* < 0.05) among treatments; data values are mean ± SD.

As shown in Figure 3A–F, L1 more closely resembled CK in maintaining plant cell membrane integrity and antioxidant defence systems, and significantly outperformed L2. For MDA, after treatment for 10, 20, and 30 d, the MDA content in L1 was significantly lower than that in L2, and showed no difference compared to CK (Figure 3A). For CAT (Figure 3B) and SOD (Figure 3D), within the treatment period, no significant difference was observed between L1 and CK, but both were significantly higher than L2. GR activity was significantly higher in L1 than in L2 after treatment for 20 and 30 d (Figure 3E). Concurrently, PAL activity, associated with stress-responsive secondary metabolism, was also highest in L1 at all cultivation periods (Figure 3F). In summary, compared to L2, L1 significantly maintains plant antioxidant capacity by synergistically and efficiently activating key enzyme systems, thereby effectively inhibiting membrane lipid peroxidation processes (low MDA content).

### 3.4. Effects of Low Light on Leaf Permeability Regulators and Antioxidant Substances

**Figure 4:**
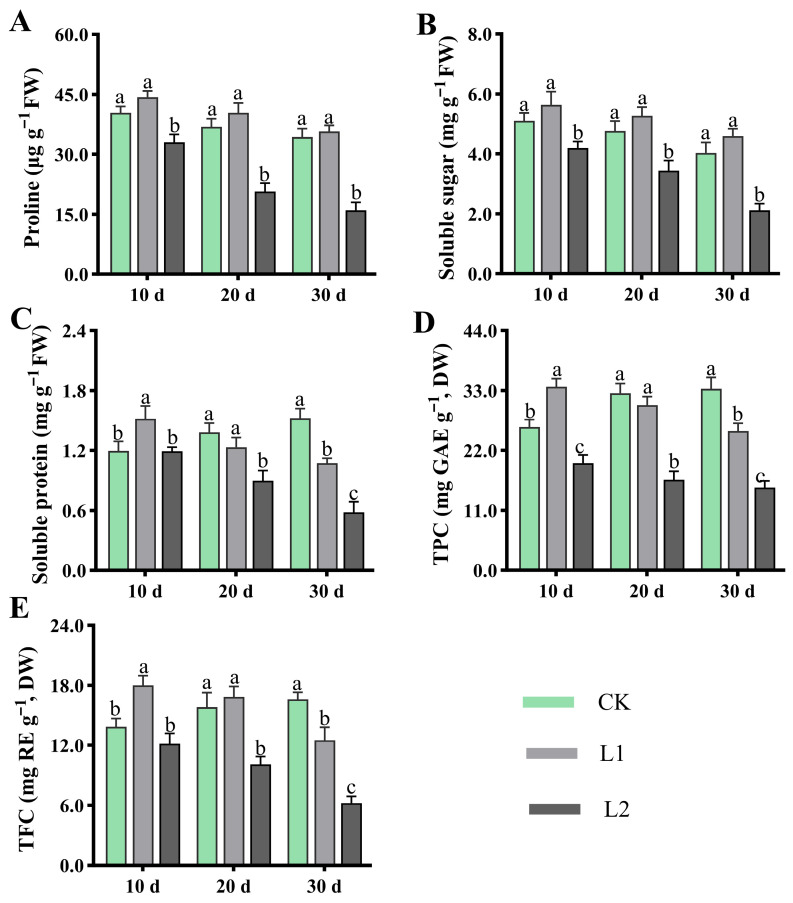
Effects of low light on *H. macrophylla* leaf permeability regulators and antioxidant substances. (**A**) Proline content. (**B**) Soluble sugar content. (**C**) Soluble protein content. (**D**) Total phenolic content (TPC). (**E**) Total flavonoid content (TFC). Different lowercase letters denote statistically significant differences (*p* < 0.05) among treatments; data values are mean ± SD.

As shown in Figure 4A–E, L1 can regulate plant osmotic balance and promote protective secondary metabolite accumulation, with physiological performance approaching or slightly exceeding that of CK, yet significantly exceeding that of L2. Proline (Figure 4A) and the soluble sugar content (Figure 4B) consistently showed higher levels in L1 than L2. The soluble protein content (Figure 4C), while initially higher than CK, decreased over the treatment period to become lower than CK, yet remained higher than L2. For protective secondary metabolite accumulation, the total phenolic content (Figure 4D) and total flavonoid content (Figure 4E) initially exceeded that of CK (after treatment for 10 d) but subsequently fell below it (after treatment for 20 and 30 d), while remaining significantly higher than that of L2. Taken together, L1 responds to low light by synergistically maintaining elevated levels of osmotic regulatory substances (proline, soluble sugars, soluble proteins) and effectively inducing the synthesis of protective secondary metabolites (total phenolics, flavonoids).

### 3.5. Effects of Low Light on Leaf Metabolites

**Figure 5:**
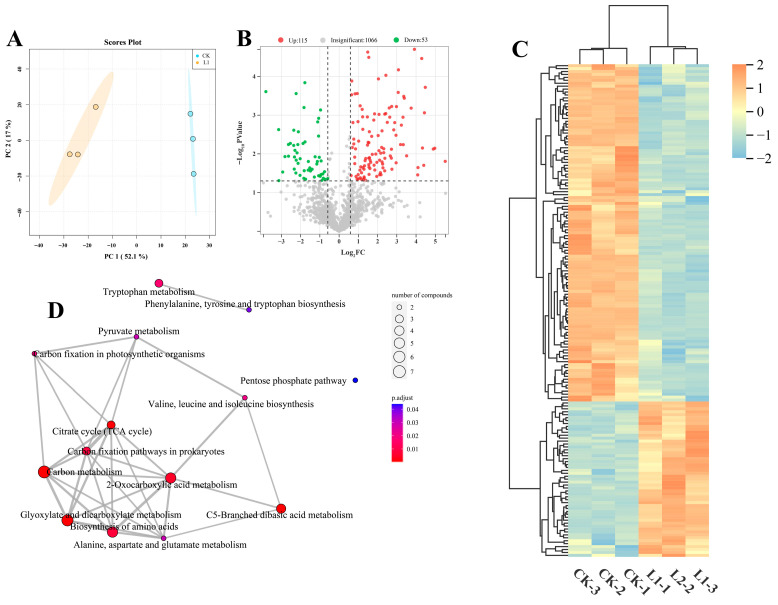
Effects of low light on *H. macrophylla* leaf metabolites after treatment for 10 d. (**A**) PCA. (**B**) Volcano plot analysis of metabolites between CK and L1 groups. (**C**) Heatmap analysis of DEMs between CK and L1 groups. (**D**) KEGG enrichment analysis between CK and L1.

Based on the results of the above indicators, samples taken 10 d after treatment were selected for transcriptomic and metabolomic analysis. Non-targeted metabolomics analysis indicates that L1 exerts a systemic effect on the plant’s metabolic profile (Figure 5). PCA results revealed a distinct separation in metabolite composition between CK and L1, with PC1 and PC2 collectively explaining 69.10% of the variance, indicating that L1 significantly remodelled the plant’s overall metabolic network (Figure 5A). Volcano plots indicate that a total of 168 significantly differentially expressed metabolites (DEMs) were identified, with 115 DEMs upregulated and 53 DEMs downregulated (Figure 5B and Table S2). Hierarchical clustering heatmaps clearly demonstrate that metabolites are distinctly grouped into several clusters exhibiting differing expression trends, with multiple clusters showing coordinated upregulation or downregulation (Figure 5C). These metabolites encompass secondary metabolites, such as amino acids, organic acids, and flavonoids. To elucidate the biological significance of these metabolic alterations, KEGG enrichment analysis was conducted. The results revealed that DEMs were significantly enriched in several core metabolic pathways, including C metabolism, TCA cycle, 2-Qxocarboxylic acid metabolism, and biosynthesis of amino acids, among others (Figure 5D). These pathways are directly linked to energy production, C metabolism, and stress-responsive secondary metabolism. Particularly noteworthy is that the enriched network reveals the TCA cycle to be closely interlinked with multiple metabolic pathways, forming a highly interconnected metabolic response module. In summary, metabolomic analysis indicates that L1 induced extensive metabolic repositioning and specifically activated synergistic metabolic networks, providing crucial insights into the molecular basis of plant physiological adaptation.

### 3.6. Effects of Low Light on Leaf Gene Expression

**Figure 6:**
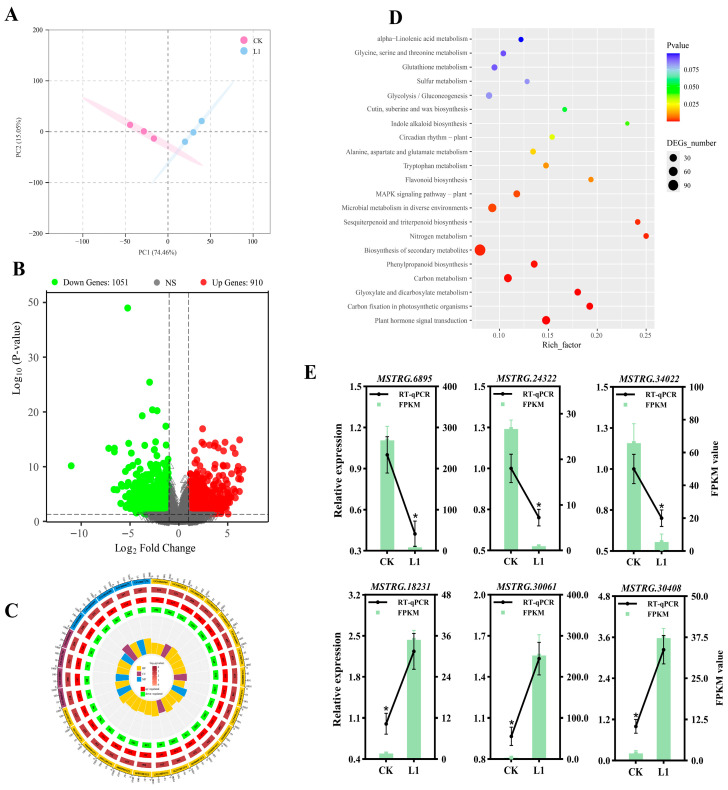
Effects of low light on *H. macrophylla* leaf gene expression after treatment for 10 d. (**A**) PCA. (**B**) Volcano plot analysis of gene expression between CK and L1 groups. (**C**) GO enrichment analysis of DEGs. (**D**) KEGG enrichment analysis between CK and L1 groups. (**E**) Validation analysis of RT-qPCR for six selected genes. The * denote statistically significant differences (*p* < 0.05) among treatments; data values are mean ± SD.

To elucidate the differences between the CK and L1 treatments at the molecular level, transcriptomic sequencing and analysis were performed (Figure 6). PCA reveals a clear separation between CK and LI samples in their overall gene expression profiles, indicating that L1 has significantly reshaped the plant’s transcriptional landscape (Figure 6A). The volcano plot clearly shows that a total of 1961 significantly differentially expressed genes (DEGs) were identified, of which 910 were upregulated and 1051 were downregulated following L1 treatment (Figure 6B and Table S3). GO enrichment analysis indicates that the DEGs are primarily enriched in biological processes such as root growth and development, tissue regeneration, and developmental regeneration; cellular components such as endosomes, cytoskeleton, and plant cell walls; and molecular functions such as transcription regulatory region DNA binding (Figure 6C). KEGG enrichment analysis indicates that the most significantly enriched pathways include carbon assimilation, energy metabolism, the synthesis of secondary metabolites, and hormone signalling, among others, which are closely associated with enhanced defence and secondary metabolism (Figure 6D). To validate the reliability of the transcriptomic sequencing data, six DEGs were randomly selected for quantitative RT-qPCR analysis (Figure 6E). The results indicate that the relative expression levels of genes detected by qPCR exhibited consistent trends with the gene expression abundances obtained from transcriptomic sequencing, suggesting that the data are valid and reliable.

**Figure 7:**
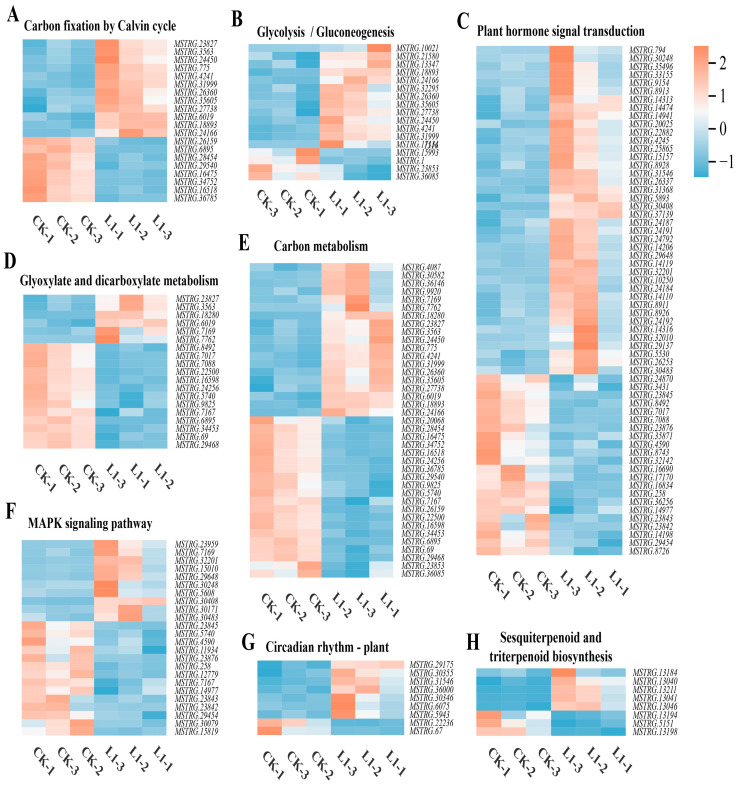
Heatmap representation of DEG expression in the top 8 KEGG enrichment pathways: carbon fixation by Calvin cycle (**A**), glycolysis/gluconeogenesis (**B**), plant hormone signal transduction (**C**), glyoxylate and dicarboxylate metabolism (**D**), carbon metabolism (**E**), MAPK signalling pathway—plant (**F**), circadian rhythm—plant (**G**), and sesquiterpenoid and triterpenoid biosynthesis (**H**).

The heatmap of DEG expression in the top eight KEGG enrichment pathways indicates that, compared with CK, L1 treatment induced systematic changes in specific pathway gene expression. In the six pathways—carbon fixation by the Calvin cycle (Figure 7A), glycolysis/gluconeogenesis (Figure 7B), plant hormone signal transduction (Figure 7C), carbon metabolism (Figure 7E), plant circadian rhythm (Figure 7G), and sesquiterpenoid and triterpenoid biosynthesis (Figure 7H)—the expression levels of a large number of relevant genes were upregulated in the L1-treated group. In contrast, in the glyoxylate and dicarboxylate metabolism (Figure 7D) pathway and the plant MAPK signalling pathways (Figure 7F), the number of genes with upregulated expression under L1 treatment was reduced. This indicates that moderate low-light stress specifically activated these gene networks associated with carbon assimilation, energy metabolism, the synthesis of secondary metabolites, and hormone signalling.

### 3.7. Integrated Transcriptomic, Metabolomic, and Physiological Analysis

**Figure 8:**
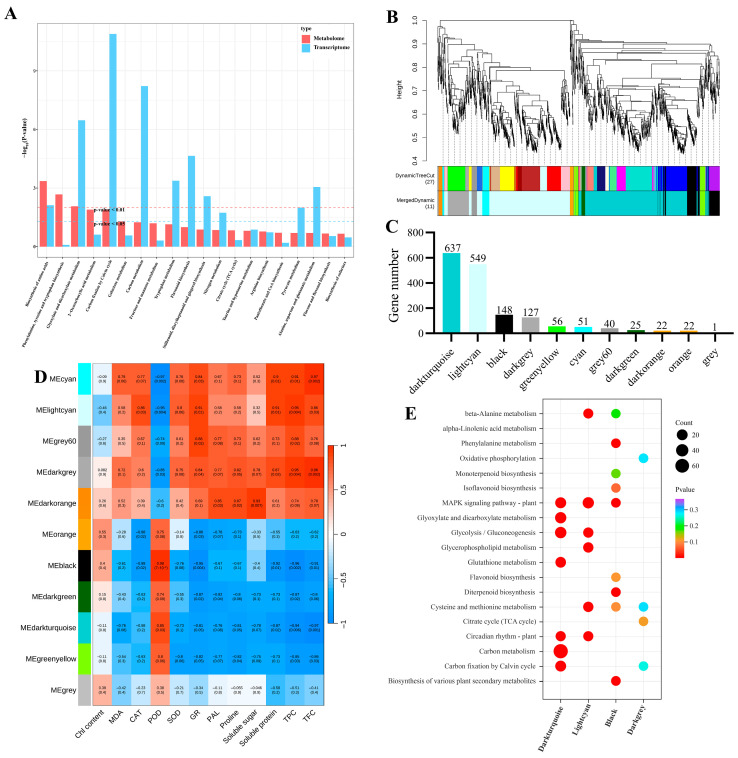
Integrated transcriptomic, metabolomic, and physiological analysis. (**A**) KEGG pathway enrichment analysis of co-expression DEMs and DEGs. (**B**) Gene clustering dendrogram and classification of 11 gene modules. (**C**) Number of genes corresponding to the 11 gene modules. (**D**) Correlation analysis of 11 gene modules with relevant physiological indicators. (**E**) KEGG enrichment analysis of genes enriched in the dark turquoise, light cyan, black, and dark grey modules.

Firstly, this study integrated KEGG pathway analysis to correlate DEGs and DEMs, aiming to elucidate the mechanisms underlying the interactions between gene expression and metabolites. The results indicated that the biosynthesis of amino acids, glyoxylate and dicarboxylate metabolism, carbon fixation by the Calvin cycle, flavonoid biosynthesis, and carbon metabolism pathways were significantly enriched (Figure 8A). Additionally, weighted gene co-expression network analysis (WGCNA) was performed to examine the correlation between DEGs and relevant physiological indicators. After filtering out genes with low expression variability via WGCNA analysis from the 1951 DEGs, the remaining 1678 genes were assigned to 11 co-expression clusters (Figure 8B,C). Correlation analysis between physiological indicators and modules revealed that GR activity, MDA, proline, soluble sugars and proteins, total phenols, and total flavonoids were significantly positively correlated with light cyan and negatively correlated with dark turquoise (Figure 7D). KEGG enrichment analysis indicated that genes in these modules are primarily involved in pathways such as C metabolism, glycolysis/gluconeogenesis, glutathione metabolism, MAPK signalling pathway—plant, etc. Based on these results, it is hypothesised that these pathway alterations significantly influence corresponding physiological indicator changes.

**Figure 9:**
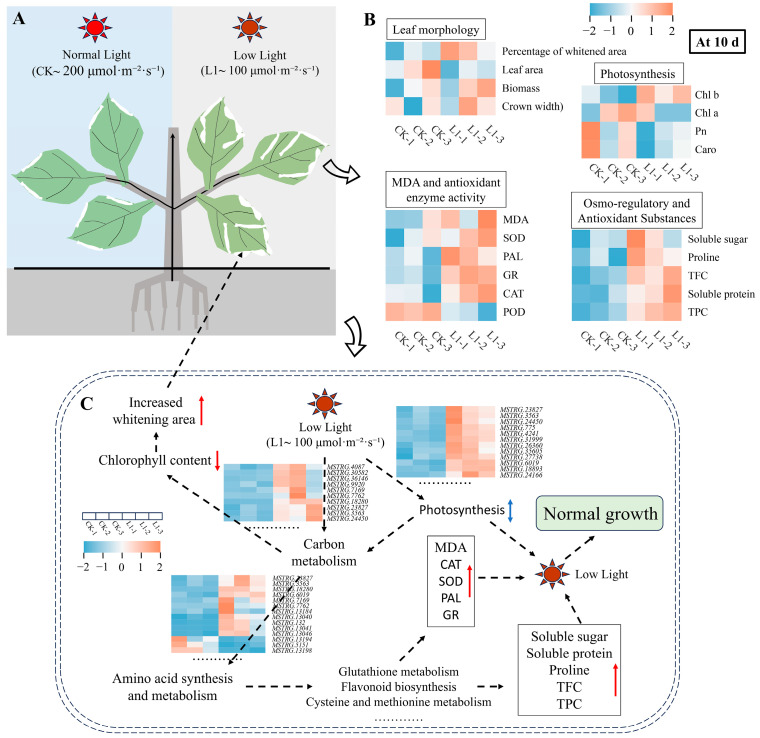
Integrated analysis of transcriptomic, metabolomic, and physiological parameters under normal and low-light treatments. (**A**): Plant phenotypes under different light treatments. (**B**) Heat map analysis of key physiological indicators 10 d after treatment. (**C**) Schematic diagram of pathways involved in the response to low-light treatments.

Based on the analysis of the above results, the mechanism by which plants adapt to L1-induced ornamental whitening under low light conditions has been elucidated. As shown in Figure 9, L1 treatment significantly increased white-bleached leaf area in *H. macrophylla* while maintaining plant growth and net photosynthetic rates comparable to those under CK light conditions. The underlying mechanism may be related to the L1 treatment synergistically activating the antioxidant enzyme system, which promotes the accumulation of osmoregulatory and protective substances such as proline, soluble sugars, and total phenolics, thereby effectively mitigating lipid peroxidation damage to cell membranes. Related pathways, such as carbon metabolism, amino acid metabolism, and glutathione metabolism, are specifically regulated and collectively form the basis of the plant’s systemic adaptation for maintaining physiological homeostasis under LI treatment.

## 4. Discussion

By integrating physiological, metabolic, and transcriptomic analyses, this study systematically elucidates the underlying mechanisms by which moderate low light (L1, 100 μmol·m^−2^·s^−1^) induces an increase in white-coloured regions in *H. macrophylla* leaves to enhance their ornamental value, while simultaneously maintaining their overall physiological functions. The results show that while L1 treatment slightly induced leaf whitening, there were no significant differences in growth indices such as crown width, biomass, and leaf area compared to CK. It is thus suggested that the whitening induced by L1 may represent an active, adaptive resource reallocation strategy [9,11,26,27]. Under limited light conditions, plants may reduce chloroplast development or synthesis in certain leaf regions (manifesting as whitening), thereby concentrating limited resources (e.g., nitrogen, carbon, and energy) more effectively on the remaining healthy green tissue to sustain efficient photosynthetic function [10,26]. This explains why the Pn, Gs, and chlorophyll content remained at levels close to those of CK following L1 treatment (Figure 1). Furthermore, L1 increases the content of carotenoids, which serve not only as photosynthetic accessory pigments but also as important photoprotective compounds, promoting endogenous antioxidant enzyme activity and scavenging excess ROS [4,28,29,30]. It is speculated that plants sensed potential photorestriction risks under L1 conditions (i.e., energy imbalance between photosystems under low-light conditions may also generate ROS) and enhanced their photoprotective capacity in advance.

Furthermore, the results regarding the antioxidant system indicate that the L1 treatment effectively activated the antioxidant enzyme system, with SOD, POD, CAT, and GR activity all significantly higher than in L2, and comparable to or even higher than in CK. Meanwhile, the membrane lipid peroxidation product MDA content remained at a low level in L1, showing no difference from CK (Figure 3). The above findings indicate that L1 induced an efficient, synergistic ROS scavenging network, keeping oxidative damage at a low level [31,32,33]. Consequently, the accumulation patterns of osmotic regulators (proline, soluble sugars, soluble proteins) and antioxidant secondary metabolites (total phenols, total flavonoids) in L1 further support this view (Figure 4). These substances not only maintained the cellular osmotic balance but also inherently possessed potent free radical scavenging capabilities [15,34,35,36]. Consequently, the antioxidant and osmotic regulation capabilities of plants under L1 treatment were sufficient to cope with low-light stress, thereby avoiding the oxidative damage and physiological disturbances observed in L2.

Multi-omics analysis provides a molecular-level explanation for the above physiological phenomena. Transcriptomic analysis revealed that L1 treatment significantly reshaped the plant’s gene expression profile, with DEGs primarily enriched in key pathways such as the Calvin cycle, carbon metabolism, secondary metabolite biosynthesis, etc. (Figure 6). This finding suggests that L1 treatment coordinates the resource allocation between carbon assimilation, nitrogen metabolism, and defence compound synthesis at the molecular level [37,38,39]. The heatmap of gene expression in key pathways further reveals that numerous genes are upregulated in pathways related to energy and carbon skeleton supply, such as the Calvin cycle, glycolysis/gluconeogenesis, and carbon metabolism. This may be an adapted response to maintain basic metabolic flux as much as possible under low-light conditions, thereby providing substrates for defence responses. Concurrently, the widespread upregulation of genes involved in phytohormone signalling pathways suggests that multiple phytohormone signals are involved in this systemic adaptive response, coordinating the adjustment of the growth–defence balance following stress perception [40,41,42,43]. Metabolomic analysis further confirmed and expanded upon the functional implications of the aforementioned transcriptional changes, with DEMs showing significant enrichment in key pathways such as carbon metabolism, TCA cycle, 2-oxocarboxylic acid metabolism, amino acid biosynthesis, etc. Among these, the synergistic upregulation of the TCA cycle and amino acid metabolism ensures that basic energy (ATP) and carbon skeleton supplies are maintained even under low-light conditions, thereby supporting the metabolic demands of biosynthesis and stress responses [44,45,46]. Meanwhile, the activation of the phenylpropanoid/flavonoid biosynthetic pathways directly drives the accumulation of antioxidant secondary metabolites such as total phenolics and total flavonoids [41,42,47,48].

However, this study has certain limitations that will serve as future directions for in-depth exploration. Firstly, the multi-omics analysis is based on a single time point. Although 10 d represents a critical response period, future dynamic analyses across multiple time points will provide a more comprehensive explanation of the adaptive mechanisms. Additionally, no spatially resolved cytological or biochemical analyses of the whitened and green tissues have been conducted, which will form the focus of future studies aimed at validating the hypothesis of resource reallocation and clarifying the cytological nature and metabolic state of the whitened regions.

## 2. Materials and Methods

### 2.1. Experimental Materials and Design

Healthy, uniformly growing annual *H. macrophylla* plants were selected and planted in a potting mix comprising nutrient-rich soil, perlite and vermiculite at a ratio of 5:3:2. Then, after 5 d of adaptation in an artificial climate chamber (light intensity 200 μmol·m^−2^·s^−1^, temperature 25 °C, humidity 70%), the plants were placed under low-light conditions for subsequent experiments. The experiment consisted of three treatments: CK (200 μmol·m^−2^·s^−1^), moderate low light L1 (100 μmol·m^−2^·s^−1^), and severe low light L2 (20 μmol·m^−2^·s^−1^). Each treatment comprised three replicates, with six plants per replicate, and the experimental unit in this study is a single plant. All subsequent parameter measurements were based on these three independent biological replicates. The plants were watered once every 2 d, and their physiological indicators were detected every 10 d, with the treatment lasting for 30 d in total.

### 2.2. Growth Indicator Detection

The entire plant morphology was photographed and recorded using a digital camera; crown width was measured using a tape measure (1 mm); and leaf area and proportion of whitened area were measured using ImageJ software (http://imagej.org, Java 1.8.0_345 (64-bit)). After washing the roots, stems, and leaves with deionised water to remove surface impurities, dry them in an oven at 80 °C for 72 h to obtain whole plant biomass (DW) [49].

### 2.3. Photosynthetic Indicator Detection

From 9:00 to 12:00, the net photosynthetic rate (Pn), stomatal conductance (Gs), and transpiration rate (Tr) were monitored using a portable photosynthesis measurement system (CIRAS-3, PP SYSTEMS, Amesbury, MA, USA). Program settings: PAR at 1000 μmol m^−2^ s^−1^, leaf chamber temperature 25 °C, reference CO_2_ concentration set to ambient, flow rate 250 mL min^−1^, relative humidity 60%. Using the same leaves, chlorophyll fluorescence was measured using an FMS-2 pulse-modulated fluorometer (PP SYSTEMS, Amesbury, MA, USA) to calculate the maximum photochemical efficiency of PSII (Fv/Fm), PSII photochemistry effective quantum yield (ΦPSII), photochemical quenching (qP), nonphotochemical quenching (qN), and PSII photosynthetic electron transport rate (ETR). On the same day, chlorophyll and carotenoid contents were determined using the same leaves as above, following the method of Duan et al. [50]. At least 12 leaves were analysed per treatment.

### 2.4. Malondialdehyde and Antioxidant Enzyme Activity Detection

MDA contents and antioxidant enzyme activity were measured using assay kits purchased from Beijing Solarbio Science & Technology Co., Ltd. (Beijing, China) in accordance with the relevant literature [22,33]. Specifically, take the aforementioned 0.1 g leaf sample, chop it finely, and place it in a mortar. Grind it into a powder using liquid nitrogen, then add 1 mL of extraction solution. Vortex thoroughly for 3–5 min, then centrifuge at 8000× *g* for 10 min at 4 °C. Following the manufacturer’s instructions, transfer the supernatant to ice and measure the absorbance values for malondialdehyde (MDA: 450, 532, 600 nm), catalase (CAT: 405 nm), peroxidase (POD: 470 nm), superoxide dismutase (SOD: 560 nm), glutathione reductase (GR: 340 nm), and phenylalanine ammonia lyase (PAL: 290 nm). MDA and antioxidant enzyme activity (CAT, SOD, POD, GR, and PAL) units are denoted as nmol g^−1^ FW and U g^−1^ FW, respectively, with six replicates per treatment.

### 2.5. Soluble Sugar, Soluble Protein, and Proline Content Detection

Referencing the method reported by Gong et al. [23] and Gandonou et al. [51], the contents of proline, soluble sugars, and soluble proteins were determined using the ninhydrin colorimetric assay, the Coomassie brilliant blue colorimetric method, and the anthrone method, respectively. The plant samples tested were sourced from Section 2.3, with substance extraction referenced in the literature. Measure the absorbance values of proline, soluble sugars, and soluble proteins at 520 nm, 630 nm, and 595 nm, respectively. Results are expressed as mg g^−1^, FW, with six replicates per treatment.

### 2.6. Total Phenolic and Total Flavonoid Content Detection

The extraction method follows Tao et al. [52] with minor modifications. Briefly, weigh 0.5 g of leaves derived from 2.3 into a 10 mL centrifuge tube and add 8 mL of 85% methanol. Place in an ultrasonic bath at 50 °C for 45 min, then centrifuge at 5000 r/min for 10 min and collect the supernatant. After repeating the extraction twice, combine the supernatants and dilute to a final volume of 25 mL for subsequent determination. The determination of total phenolic and total flavonoid content was performed using the Folin–Ciocalteu method and the Al(NO_3_)_3_ colorimetric method, detecting absorbance values at 765 nm and 500 nm, respectively. Total phenolic and total flavonoid results were expressed as gallic acid equivalents (mg GAE/g DW) and rutin equivalents (mg RE/g DW), respectively, with six replicates per treatment.

### 2.7. Metabolomics

Three independent biological replicates of leaf samples were collected from each treatment (CK and L1) for metabolomic analysis. The specific procedure is as follows: (1) Weigh 100 mg of CK and L1-treated leaf samples ground in liquid nitrogen into an EP tube, and add 500 μL of 80% methanol aqueous solution. (2) Vortex briefly, ice-bathe for 5 min, and then centrifuge at 15,000× *g*, 4 °C for 20 min. (3) Dilute a portion of the supernatant with mass spectrometry-grade water to achieve a methanol concentration of 53%. (4) Centrifuge at 15,000× *g*, 4 °C for 20 min, collect the supernatant, and inject for LC-MS analysis. UHPLC-MS/MS analyses were performed using a Vanquish UHPLC system (ThermoFisher, Dreieich, Germany) coupled with an Orbitrap Q ExactiveTMHF-X mass spectrometer (Thermo Fisher, Dreieich, Germany). Samples were injected onto a Hypersil Gold column (100 × 2.1 mm, 1.9 μm) at a flow rate of 0.2 mL/min. The eluents for the positive and negative polarity modes were eluent A (0.1% FA in Water) and eluent B (Methanol). The solvent gradient was set as follows: 2% B, 1.5 min; 2–85% B, 3 min; 85–100% B, 10 min; 100-2% B, 10.1 min; 2% B, 12 min. The Q ExactiveTM HF mass spectrometer was operated in positive/negative polarity mode with a spray voltage of 3.5 kV, capillary temperature of 320 °C, sheath gas flow rate of 35 psi, aux gas flow rate of 10 L/min, S-lens RF level of 60, and aux gas heater temperature of 350 °C. The raw data have been uploaded to the MetaboLights database (the relevant link is https://www.ebi.ac.uk/metabolights/reviewera3cb2b15-d936-4005-b602-0f2e96d2de4c, accessed on 29 March 2026). Subsequent metabolite identification and quantification were processed and analysed by Wekemo Tech Co., Ltd., Shenzhen, China. Differential metabolites (DEMs) were screened under the conditions of log_2_|FoldChange| > 1, VIP > 1, and *p* < 0.05. These DEMs were annotated using the KEGG database (https://www.genome.jp/kegg/pathway.html, accessed on 29 March 2026), HMDB database (https://hmdb.ca/ metabolites, accessed on 29 March 2026), and LIPIDMaps database (http://www.lipidmaps.org/, accessed on 29 March 2026). PCA, volcano plots, clustering heatmaps, and KEGG enrichment analysis were conducted in Metware Cloud (https://cloud.metware.cn, accessed on 29 March 2026) and Mepaboanalyst (https://www.metaboanalyst.ca, accessed on 29 March 2026; version 6.0).

### 2.8. Transcriptomics

Three independent biological replicates of leaf samples were collected from each treatment (CK and L1) for metabolomic analysis. The specific detection and analysis procedures are as follows. The extraction and detection of RNA from CK and L1-treated leaf samples (using the RNAprep Pure Polysaccharide-Polyphenol Plant Total RNA Extraction Kit DP441), library construction and quality control, and sequencing testing were performed by Wekemo Tech Co., Ltd., Shenzhen, China. The raw data has been uploaded to the NCBI database (relevant link https://dataview.ncbi.nlm.nih.gov/object/PRJNA1444700?reviewer=j0q3rqq1hr4g2vd45koov3k584, accessed on 29 March 2026). To ensure the quality and reliability of data analysis, quality control processing was performed on the raw data using the fastp software (0.23.1). The specific procedures are as follows: (1) Remove adapter sequences from the reads and discard any reads that contain no inserted fragments due to adapter self-ligation or similar causes. (2) Trim bases at the 3’ end of the sequence that have low quality (quality score less than 20); if the remaining sequence still contains bases with a quality score less than 10, discard the entire sequence; otherwise, retain it. (3) Remove reads with an N-content exceeding 10%. (4) Discard sequences shorter than 75 bp after adapter removal and quality trimming. Concurrently, perform Q20, Q30, and GC content calculations on the clean data. Perform alignment analysis of the cleaned data with the reference genome (https://www.ncbi.nlm.nih.gov/datasets/genome/?taxon=23110, GCA_033977485.1_Hmc_EndlessSummer_p1.0_genomic.fna, accessed on 29 March 2026). The FPKM method was employed to compare expression level differences between sample genes, while DESeq2 software (1.26.0) was utilised for differential expression analysis between samples. The screening criteria for differentially expressed genes (DEMs) were set at |log_2_ (FoldChange)| > 1 and *p* < 0.05, and are presented in Supplementary Files (Table S2). PCA, volcano plots, GO, and KEGG analyses were conducted on websites https://www.bioincloud.tech/pipelines and https://cloud.metware.cn (accessed on 10 April 2026).

### 2.9. RT-qPCR Validation

Subsequently, genes exhibiting significant differences were randomly selected for qRT-PCR analysis to validate the reliability of the transcriptomic data. Primer sequences for the actin gene and the six target genes mentioned above were designed and synthesised by Sangon Biotech (Shanghai, China) Co., Ltd., with specific details provided in Table S1. The qRT-PCR analyses were performed using the Rad CFX96™ system (Bio-Rad, Hercules, CA, USA) and SYBR Green Premix Pro Taq HS premixed qPCR kit. Experimental procedures and instrument settings for the qRT-PCR analysis were based on the methodology described in [53]. The actin gene was used as an internal control to normalise the data, and the relative expression levels of the genes were calculated using the 2^−∆∆Ct^ values. Three biological replicates were used for each treatment.

### 2.10. Statistical Analysis

Statistical analysis was performed with SPSS (version 25.0; IBM Corp., Armonk, NY, USA). Group differences were assessed by one-way ANOVA, with post hoc comparisons conducted using Tukey’s test (*p* < 0.05). Data are expressed as the mean ± SD. The figures were generated using Origin 2024 (OriginPro, Northampton, MA, USA). Heatmaps of DEGs and DEMs were visualised on the MetWare Cloud platform (https://cloud.metware.cn, accessed on 29 March 2026).

## 5. Conclusions

This study indicates that moderate low light (L1, 100 μmol·m^2^·s^−1^) induces enhanced leaf whitening in *H. macrophylla*, while maintaining normal plant growth. Specifically, although L1 treatment reduced chlorophyll synthesis in some leaves (leading to whitening), it systematically maintained the plants’ photosynthesis, antioxidant enzyme activity (SOD, GR), and secondary metabolite synthesis (phenolics, flavonoids). Transcriptomic and metabolomic analyses have further revealed that this physiological adaptation involves specific molecular reprogramming across multiple pathways, including energy metabolism and the synthesis and metabolism of defence compounds. This study, from the perspective of stress aesthetics, offers new scientific insights and technical potential for the precise regulation of ornamental plant phenotypes through abiotic stress.

## Figures and Tables

**Table 1:** Effects of low light on Fv/Fm, ΦPSII, qP, qN and ETR of *H. macrophylla*.

Time (d)	Treatments	Fv/Fm	ΦPSII	qP	qN	ETR
10 d	CK	0.85 a	0.68 a	0.83 a	0.42 c	108.53 a
	L1	0.82 a	0.61 b	0.71 b	0.56 b	110.46 a
	L2	0.53 b	0.30 c	0.42 c	0.79 a	53.54 b
20 d	CK	0.83 a	0.65 a	0.85 a	0.39 c	111.58 a
	L1	0.79 a	0.56 a	0.69 b	0.53 b	103.25 b
	L2	0.47 b	0.26 b	0.38 c	0.77 a	41.46 c
30 d	CK	0.86 a	0.65 a	0.84 a	0.40 c	115.78 a
	L1	0.77 b	0.52 a	0.63 b	0.59 b	92.47 b
	L2	0.43 c	0.22 b	0.36 c	0.78 a	35.61 c

Different lowercase letters denote statistically significant differences (*p* < 0.05) among treatments; all values are represented as mean (n = 12).

## Data Availability

The original contributions presented in this study are included in the article/Supplementary Materials. Further inquiries can be directed to the corresponding author.

## Supplementary Materials

The following supporting information can be downloaded at: https://www.mdpi.com/article/10.3390/genes17050545/s1.
